# Genome editing in maize directed by CRISPR–Cas9 ribonucleoprotein complexes

**DOI:** 10.1038/ncomms13274

**Published:** 2016-11-16

**Authors:** Sergei Svitashev, Christine Schwartz, Brian Lenderts, Joshua K. Young, A. Mark Cigan

**Affiliations:** 1 Trait Enabling Technologies, DuPont Pioneer, Johnston, Iowa 50131, USA

## Abstract

Targeted DNA double-strand breaks have been shown to significantly increase the frequency and precision of genome editing. In the past two decades, several double-strand break technologies have been developed. CRISPR–Cas9 has quickly become the technology of choice for genome editing due to its simplicity, efficiency and versatility. Currently, genome editing in plants primarily relies on delivering double-strand break reagents in the form of DNA vectors. Here we report biolistic delivery of pre-assembled Cas9–gRNA ribonucleoproteins into maize embryo cells and regeneration of plants with both mutated and edited alleles. Using this method of delivery, we also demonstrate DNA- and selectable marker-free gene mutagenesis in maize and recovery of plants with mutated alleles at high frequencies. These results open new opportunities to accelerate breeding practices in a wide variety of crop species.

## Abstract (web summary)

Genome editing in plants typically requires the expression of Cas9 and guide RNA from stably transformed plasmid DNA. Here, the authors show that successful editing can be achieved after delivery of the Cas9-guide RNA complex as a ribonucleoprotein to maize embryos via biolistics.

## Graphical abstract

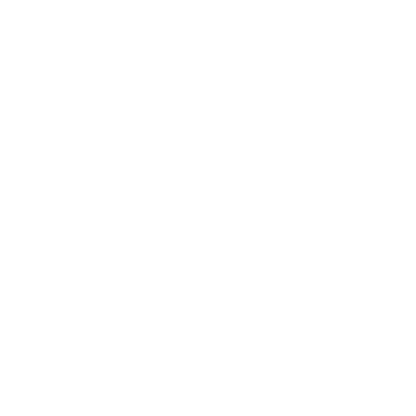

Demonstration that targeted DNA double-stranded breaks (DSBs) increase the frequency of gene editing by about 1,000 fold was a fundamental breakthrough in the field of genome modification^1^,^2^,^3^. DSBs in eukaryotic cells are repaired using two major pathways: non-homologous end joining (NHEJ) and homology-directed repair^4^. NHEJ may lead to imperfect repair resulting in a range of different mutations. In contrast, homology-directed repair, although less frequent in somatic cells, can promote precise gene editing and site-specific gene insertion by utilizing exogenously supplied repair DNA templates containing the sequence of interest flanked by regions of homology flanking the DSB site. In the past two decades, several technologies capable of generating targeted DSBs have been developed, including zinc-finger nucleases, customized homing endonucleases (meganucleases), transcription activator-like effector nucleases (TALENs), and CRISPR-associated (Cas) proteins^5^,^6^. CRISPR–Cas has quickly become the technology of choice for most genome editing applications due to its simplicity, efficiency and versatility^7^.

In the past 4 years, RNA-guided *Streptococcus pyogenes* Cas9 endonuclease has been successfully used for genome modification in multiple plant species^8^,^9^. In the majority of these experiments, the guide RNA (gRNA), as well as the Cas9 and selectable marker genes, have been delivered into plant cells using either T-DNA (*Agrobacterium tumefaciens* infection) or plasmid DNA (particle bombardment). In both cases, the delivered DNA frequently integrates into the genome leading to various side effects such as gene disruption, plant mosaicism and potential off-site cutting^10^,^11^. Furthermore, DNA molecules often integrate into the targeted DSB sites, decreasing the efficiency of gene editing and gene insertion^11^,^12^. To mitigate these negative effects, plants with pre-integrated Cas9 nuclease have been generated and used for delivery of gRNA in the form of RNA molecules^11^. Although successful, this approach requires time and resources for development and characterization of pre-integrated lines.

DNA-free genome editing has been demonstrated in cultured human cells using electroporation-mediated delivery of gRNA and Cas9 in the form of *in vitro* assembled ribonucleoproteins (RNPs)^12^,^13^,^14^. However, in plants, the presence of a cell wall makes it impossible to use transfection or electroporation for nucleic acid and/or protein delivery. Recently, plant protoplasts, generated by the removal of this cell wall using enzymatic digestion, have been successfully used for RNP delivery and genome editing in a variety of plants such as tobacco, *Arabidopsis*, lettuce, rice and *Petunia*^15^,^16^. However, for the majority of monocotyledon species, including major crops like maize, wheat, rice, barley and sorghum, regeneration of plants from protoplasts remains either unattainable or inefficient^17^,^18^. Recently, transgene-free genome editing has been reported in wheat^19^. The authors demonstrated efficient gene mutagenesis in plants using transiently expressed CRISPR–Cas9 DNA and by delivering Cas9 and gRNA as *in vitro* transcribed RNA molecules.

In this report, we demonstrate that Cas9 and gRNA in the form of RNP complexes can be delivered into maize embryo cells via particle bombardment. The resulting regenerated plants contained specifically targeted gene mutations and gene edits at high frequencies. We also demonstrate a completely DNA- and selectable marker-free method for the recovery of plants with mutated alleles at high frequencies. To our knowledge, this is the first report demonstrating DNA-free genome editing in a major crop species using biolistically delivered Cas9–gRNA RNPs.

## Results

### Biolistic delivery of Cas9–gRNA RNP into maize cells

Previously, we demonstrated that maize genes could be mutagenized and edited using Cas9 and gRNA delivered on DNA vectors^11^,^20^. In this report, the same four genomic regions, liguleless1 (*LIG*), acetolactate synthase (*ALS2*) and two male fertility genes (*MS26* and *MS45*), were targeted by purified Cas9 protein pre-assembled with *in vitro* transcribed gRNAs. Cas9–gRNA RNP complexes were delivered into maize immature embryo cells on gold particles (0.6 μm) using a helium gene gun. Embryos bombarded with Cas9 protein alone were used as negative controls, while DNA vectors encoding Cas9 and the four gRNAs served as positive controls. In these experiments, DNA vectors encoding ‘helper genes'—cell division promoting transcription factors (maize ovule developmental protein 2 (*ODP2*) and maize Wuschel (*WUS*)^21^), and selectable and visible marker genes (*MOPAT-DSRED* fusion) were co-bombarded with the RNPs.

**Figure 1:**
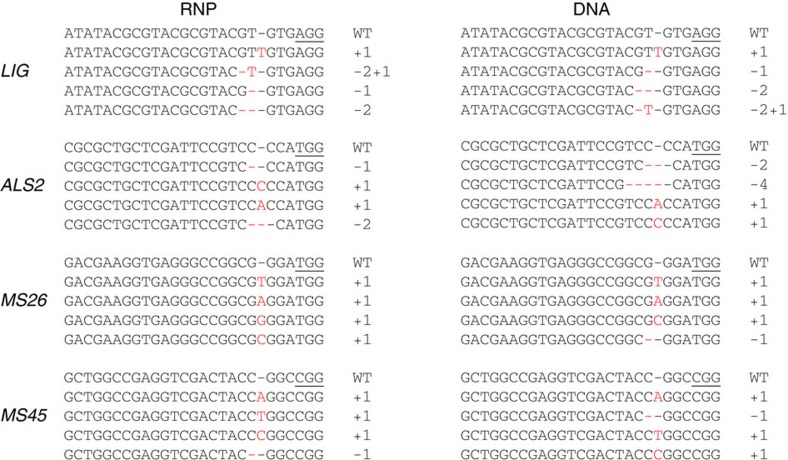
Examples of four of the most prevalent mutation types at four targeted sites generated by delivery of Cas9 and gRNAs in the form of RNPs or DNA vectors. *LIG*, *ALS2*, *MS26* and *MS45* were targeted. Protospacer adjacent motif (PAM) sequences are underlined and mutations are indicated as red dashes or letters. Black dashes indicate spaces for alignment purposes. WT indicates wild-type sequences.

To evaluate RNP delivery into maize cells and their cleavage activity, embryos were harvested 2 days after bombardment. Total genomic DNA was extracted from these harvested embryos and fragments surrounding the targeted sequences were amplified by PCR and analysed by amplicon deep sequencing. Mutations were readily detected at all target sites when Cas9–gRNA RNP complexes or Cas9 and gRNA DNA vectors were delivered, but not in negative controls (Table 1). Similar mutation frequencies with DNA and RNP delivery were observed in multiple experiments, demonstrating efficient delivery and high cleavage activity by the RNPs. Moreover, the types of mutations observed with RNP and DNA delivery experiments were nearly identical (Fig. 1).

To assess the potential of RNP delivery to reduce off-target cleavage in maize, an off-target site for the MS45–gRNA was identified as described in the methods section. The mutation frequency at the off-site was then evaluated in DNA and RNP delivery experiments. As shown in Table 1, RNP off-site activity was greatly reduced relative to that observed with Cas9 and gRNA delivery on DNA vectors. This result is consistent with previous reports in human and plant systems^12^,^13^,^14^.

To measure the mutation frequency at the plant level, 60 embryos co-bombarded with Cas9–MS45–gRNA complex, ‘helper genes' and *MOPAT-DSRED* were placed on media containing bialaphos as selective agent. Multiple plants were regenerated from each of the 36 herbicide-resistant callus sectors and screened for mutations. Out of the 36 events, 17 (47%) contained mutant alleles (10 single and 7 biallelic) while 19 (53%) revealed only wild-type *MS45* alleles. Among plants with mutations, the number of sequencing reads for each allele was similar, indicating plants were not chimeric. These results are in agreement with mutation frequencies (46%) obtained for lettuce T0 plants regenerated from protoplasts transfected with RNP complexes^15^.

### RNP delivery facilitates targeted ALS2 gene editing

**Figure 2:**
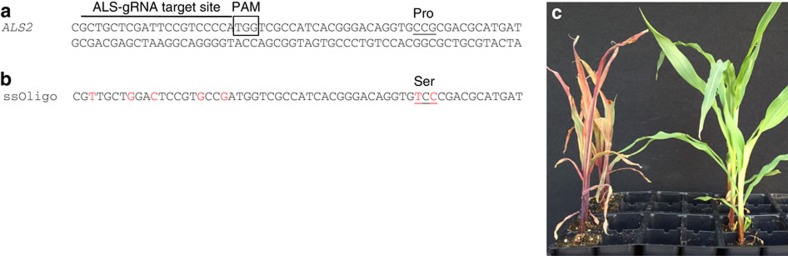
*ALS2* gene editing using co-delivery of RNP complex and single-stranded (ss) oligo as repair template. (**a**) Partial *ALS2* sequence with ALS–gRNA target site, protospacer adjacent motif (PAM) is boxed and proline codon (amino acid position 165) to be edited is underlined. (**b**) Partial sequence of the ssDNA oligo used as a repair DNA template; modified nucleotides are shown in red font and serine codon is underlined. (**c**) Wild type (left) and *ALS2* edited (right) plants tested for resistance to chlorsulfuron (200 mg l^−1^); shown at 10 days after spraying.

To demonstrate that RNP delivery can enable endogenous gene editing, one of the two maize acetolactate synthase genes (*ALS2*) was modified to introduce a single amino acid residue change: proline to serine at position 165 (Fig. 2a). This amino acid change has been shown to confer whole plant resistance to chlorsulfuron, a sulfonylurea class of herbicides^11^. A 127 nt single-stranded DNA oligo was used as repair DNA template (Fig. 2b) and co-bombarded with Cas9–ALS–gRNA RNP complex and ‘helper genes'. In two independent experiments, 40 to 50 bombarded embryos were transferred to plates containing 100 p.p.m. of chlorsulfuron as direct selection for an edited *ALS2* gene. Six weeks later, two callus sectors (one from each experiment) that continued growing on media with chlorsulfuron were analysed by sequencing. In both events, one *ALS2* allele was specifically edited while the second allele remained wild type. Plants regenerated from these callus sectors contained edited *ALS2* alleles and were resistant to chlorsulfuron when sprayed with the herbicide (Fig. 2c).

### DNA-free and selectable marker-free mutagenesis in maize

The necessity of selectable markers that provide a growth advantage to transformed cells has been the long standing paradigm in plant transformation. In both mutation and gene editing experiments described above, selectable markers (*MOPAT-DSRED* or modified endogenous *ALS2*) were used to select for genome edited events. Taking into consideration the high activity of RNP complexes in our previous experiments, we attempted completely DNA-free genome editing without a selectable marker. Maize embryo cells were bombarded with Cas9–gRNA RNP complexes targeting three different genes: *LIG*, *MS26* and *MS45*. Cas9 and MS45–gRNA on DNA vectors were delivered in parallel experiments. Plants were regenerated and analysed by sequencing for targeted mutations. In all experiments, mutant plants were recovered at surprisingly high frequencies ranging from 2.4% to 9.7% (Table 2).

In addition, regenerated plants derived from MS45–gRNA delivered as either DNA vector or complexed with Cas9 protein were screened for mutations at the off-target site described above. Similar to the results presented in Table 1, DNA delivered Cas9 and gRNA resulted in detectable mutations at this off-target site (50% frequency relative to the intended site). Remarkably, no plants with off-site mutations were identified when Cas9 and gRNA were delivered as RNP complex (Table 2).

**Figure 3:**
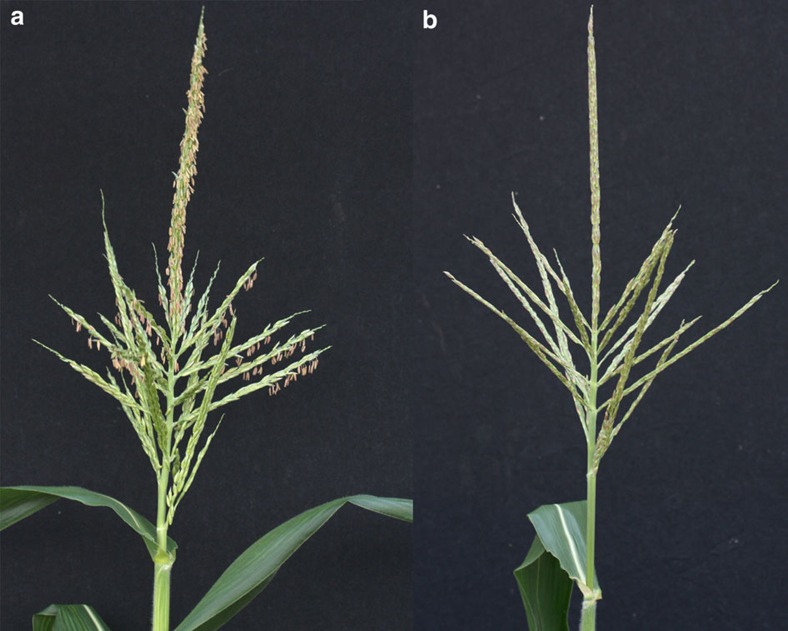
Biallelic mutations in *MS45* by RNP result in male sterile maize. (**a**) Male-fertile tassel of wild-type maize. (**b**) Male-sterile tassel of biallelic *ms45* mutant.

As shown in Table 2, and in agreement with previous reports^11^,^20^, nearly 80% of the plants containing mutations in the DNA delivery experiment were biallelic mutants. In contrast, the majority of the plants regenerated in RNP delivery experiments revealed only one mutated allele; with about 10% of plants maintaining biallelic mutations. Primary regenerated plants (T0) containing *ms45* biallelic mutations were male sterile as expected given the requirement of *MS45* for pollen development in maize^22^ (Fig. 3). T0 regenerants were crossed with wild-type Hi-II plants and the progeny were used for segregation analysis. Sexual transmission of mutated *ms45* alleles at the expected Mendelian segregation (1:1) was demonstrated in all progeny plants analysed.

## Discussion

CRISPR–Cas9 is a powerful DNA DSB technology that has wide-ranging applications in academic research, gene therapy and animal and plant breeding programs. Over the past 4 years, this technology has been successfully used to introduce genome modifications in multiple plant species, including agronomically important crops such as maize, wheat, soybean and rice^8^,^9^. Plant genome editing is limited by current transformation methods, efficiency of DNA delivery and low frequencies of plant regeneration. In contrast to human and animal systems, the presence of a thick wall surrounding every plant cell fundamentally impacts plant transformation. This wall makes it impossible to use transfection or electroporation, which are broadly used for nucleic acid and/or protein delivery in mammalian genome-editing experiments. For this reason, plant transformation primarily relies on *Agrobacterium-*mediated and biolistic delivery of CRISPR–Cas9 reagents on DNA vectors. As a result, gRNA and Cas9 expression cassettes frequently integrate into the genome and potentially lead to gene disruption, plant mosaicism and potential off-site cutting. Although these undesired secondary changes can be segregated away by several rounds of backcrossing to the wild-type parent, this can be time consuming, especially for crops with complex polyploid genomes and long breeding cycles such as soybean and wheat.

Delivery of Cas9 and gRNA in the form of RNP complexes would mitigate many of these side effects. DNA-free genome modification was first demonstrated in mammalian cell cultures^12^,^13^. In plants, delivery of meganucleases and TALENs^23^ as well as Cas9–gRNA RNP complexes has also been demonstrated by transfecting protoplasts of several species^15^,^16^. Protoplasts are ‘naked' cells generated by enzymatic removal of cell walls and are basically the equivalent of cultured mammalian and human cells. Protoplasts have a unique property of reforming the cell wall and, in some cases, regenerating into plants. Plant regeneration from protoplasts has been demonstrated for several species, including *Arabidopsis*, tobacco, *Petunia* and a few other, mostly, dicots^18^. However, despite significant effort, regeneration of fertile plants from protoplasts for the majority of crop species is highly inefficient and/or limited to specific genotypes. These current restrictions make deployment of a protoplast system as a general approach for genome editing impractical. This is the first report where Cas9–gRNA RNP complexes were biolistically delivered into maize embryo cells and resulted in regeneration of fertile plants with both mutated and edited alleles. Moreover, this approach was used to generate plants with mutated alleles in completely DNA- and selectable marker-free experiments at reasonably high frequencies.

The question of CRISPR–Cas specificity has been widely discussed in the literature^24^,^25^,^26^ with focus on human cell culture systems. DSB reagent specificity, limiting potential off-target site cleavage, is critical in any study related to human cells with potential application in gene therapy. However, in plant genome editing, even for commercial product development, potential off-site cutting is not nearly as important as long as it does not cause cytotoxicity precluding plants from normal development and reproduction. Potential off-target mutation(s) can be eliminated through several consecutive rounds of sexual reproduction (backcrossing to parental germplasm). As discussed earlier, this process can be time consuming, especially for species with large polyploid genomes, or impossible for vegetatively propagated plants such as sugarcane, potato and cassava. The frequency of off-site mutagenesis can be reduced by careful selection of a target with minimal or no potential off-target sites. It has also been demonstrated in both mammalian^12^,^13^,^14^ and plant protoplast^15^ systems that delivering RNPs instead of DNA on vectors significantly reduces the frequency of off-site cleavage. In this study, an active off-target site for MS45–gRNA was identified and evaluated for mutation frequencies in both DNA and RNP delivery experiments. In full agreement with previous reports, delivery of RNP complexes demonstrated significantly decreased mutation frequencies in comparison to DNA vectors. For both immature embryos and mature plants, Cas9 and gRNA delivered on DNA vectors demonstrated high frequency of off-site mutations (50%) when compared with the MS45 target site. Remarkably, off-site mutations were not detected in regenerated plants when RNPs were used. These results demonstrate that Cas9–gRNA delivered as RNP complex has a significant advantage over DNA vector delivery by promoting high mutation frequencies in a more precise manner.

In conclusion, the ability to deliver active Cas9–gRNA RNP complexes on gold particles into maize cells, combined with the high frequency of mutant plant recovery without selection, makes this approach practical for genome editing in major crop species. In light of recent reviews describing applications of CRISPR–Cas technology in plants^6^,^27^, the ability to deliver RNP biolistically significantly broadens options to modify the plant genome. The results presented here in maize represent a major paradigm shift for plant transformation and provide new opportunities to advance agricultural breeding practices for any plant species amenable to biolistic delivery, including other major crops like rice, wheat, soybean, barley and sorghum.

## Methods

### Plant material

Publicly available maize Hi-II line^28^ was obtained from internal DuPont Pioneer sources.

### Plasmids and reagents used for plant transformation

Cas9 gene from *S. pyogenes* M1GAS (SF370) was maize codon-optimized and included the potato ST-LS1 intron. To facilitate nuclear localization of Cas9 protein in maize cells, *Simian virus 40* (SV40) monopartite amino terminal nuclear localization signal (MAPKKKRKV) and *A. tumefaciens* bipartite VirD2 T-DNA border endonuclease carboxyl terminal nuclear localization signal (KRPRDRHDGELGGRKRAR) were incorporated at the amino and carboxyl termini of the Cas9 open reading frame, respectively (Supplementary Note 1). The maize-optimized Cas9 gene was linked to a maize constitutive Ubiquitin-1 promoter, while transcription of this gene was terminated by the addition of the 3′ sequence from the potato proteinase inhibitor II gene (PinII). To generate a single gRNA, maize U6 polymerase III promoter and terminator were isolated and used to direct initiation and termination of gRNAs, respectively. Two *Bbs*I restriction endonuclease sites were introduced in an inverted tandem orientation with cleavage orientated in an outward direction to facilitate the rapid introduction of maize genomic DNA target sequences into the gRNA expression vectors (Supplementary Note 2). Only target sequences starting with a G nucleotide were used to promote favourable polymerase III expression of the gRNA.

Plasmids containing cell division promoting transcription factors (maize ovule developmental protein 2 (*ODP2*) and maize Wuschel (*WUS*)), selectable and visible marker *MOPAT-DSRED* (a translational fusion of the bialaphos resistance gene, phosphinothricin-N-acetyl-transferase, and the red fluorescent protein *DSRED*) were previously described^29^. For *ALS2* gene editing, a single-stranded 127 nt oligo homologous to the fragment spanning the ALS2 target site with seven single nucleotide changes (Fig. 2b) was synthesized (Integrated DNA Technologies, USA). One C to T and one G to C replacements were introduced to change proline to serine at position 165 in the *ALS2* gene. SNPs within the gRNA target site sequence were also introduced to prevent Cas9 protein cleavage of the edited allele.

### Cas9 protein and gRNA molecules

*S. pyogenes* Cas9 protein with two nuclear localization signals (Supplementary Note 3) was received from the internal DuPont sources. To generate gRNA in the form of RNA molecules, the maize-optimized U6 polymerase III gRNA expression cassettes were amplified by PCR using a 5′ oligonucleotide primer that also contained the sequence of the T7 polymerase promoter and transcriptional initiation signal just 5′ of the spacer. T7 *in vitro* transcription was carried out with the AmpliScribe T7-Flash kit (Epicentre, USA) according to the manufacturer's recommendations and products were purified using phenol/chloroform extraction and NucAway Spin Columns (Life Technologies Inc., USA) followed by ethanol precipitation.

### RNP complex formation

To generate a guide RNA–Cas9 ribonucleoprotein (RNP) complex, 7 μg of Cas9 protein and 3 μg of gRNA molecules (1:2 molar ratio) were mixed in 1 × NEB Buffer 3 and 1 μl of RNA inhibitor (Ribo Guard, Epicentre, USA) to a total volume of 20 μl and incubated at room temperature for 15 min.

### Particle bombardment and plant regeneration

The particle delivery matrix comprised of the RNP complexes alone, or complemented with plasmids containing Ubiquitin promoter-regulated selectable and visible marker, *MOPAT-DSRED* fusion (125 ng), Ubiquitin promoter-regulated ODP2 (60 ng), and maize IN2 promoter-regulated WUS (60 ng) were delivered into maize embryo cells using standard particle delivery protocol^29^ with minor modifications. Briefly, the RNPs and DNA were precipitated onto 0.6 μm (average diameter) gold particles (Bio-Rad, USA) using a water soluble cationic lipid TransIT-2020 (Mirus, USA) as follows: 50 μl of gold particles (water suspension of 10 mg ml^−1^) and 2 μl of TransIT-2020 water solution were added to the premixed RNPs and DNA vectors, mixed gently, and incubated on ice for 10 min. RNP/DNA-coated gold particles were then pelleted in a microfuge at 8,000*g* for 30 s and supernatant was removed. The pellet was resuspended in 50 μl of sterile water by brief sonication. Immediately after sonication, coated gold particles were loaded onto a macrocarrier (10 μl each) and allowed to air dry. Immature maize embryos, 8–10 days after pollination, were bombarded using a PDS-1000/He Gun (Bio-Rad, USA) with a rupture pressure of 425 pounds per inch square. Post-bombardment culture, selection, and plant regeneration were performed as previously described^30^. Regenerated plantlets were moved to soil, where they were sampled and grown to maturity in greenhouse conditions.

### PCR and amplicon deep sequencing

All steps were performed as previously described^11^. Briefly, DNA was extracted by placing two leaf punches, two stainless steel beads, and 500 μl of extraction buffer (Buffer PB, Qiagen, Germany) into each tube of a microtitre rack. Samples were homogenized using a Genogrinder (Gino/Grinder 2000, SPEX SamplePrep, USA) at 1,650 r.p.m. for 1 min, followed by centrifugation at 3,500*g* for 20 min. Supernatant (300 μl) was pipetted into wells of a GF filter plate (Nunc, Denmark). Then, 600 μl of wash buffer (50 mM Tris/HCl, pH 7.4; 200 mM NaCl; 70% EtOH) was applied once and the plate was centrifuged at 3,500*g* for 10 min. DNA was eluted with 120 μl of 1:10 dilution of TE by centrifugation at 1,000*g* for 1 min.

For deep sequencing analysis, 30 Hi-II maize immature embryos were collected 2 days after bombardment, pooled, and total genomic DNA was extracted. The DNA regions surrounding the intended target sites were amplified by PCR using Phusion High Fidelity PCR Master Mix (NEB, USA) adding on the sequences necessary for amplicon-specific barcodes and Illumina sequencing using ‘tailed' primers through two rounds of PCR. The primers used in the primary and secondary PCR reactions are shown in Supplementary Tables 1 and 2. The resulting PCR amplifications were purified with a Qiagen PCR purification spin column (Qiagen, Germany), combined in an equimolar ratio, and single read 150 nt-length amplicon sequencing was performed on MiSeq Personal Sequencer (Illumina Inc.,USA). Only those reads with ≥1 nucleotide INDEL arising within the 10-nt window centred over the expected site of cleavage that were not found in the negative controls were classified as NHEJ mutations. The total number of visually confirmed mutations were used to calculate the percentage of mutant reads.

### Identification of a maize off-target site

Searches for a site with close homology to the on-target site were performed by aligning the protospacer region of the MS45 target site (the region of the target site that base pairs with the guide RNA spacer) with the maize B73 reference genome (B73 RefGen_v3, Maize Genetics and Genomics Database) using Bowtie sequence aligner^31^ permitting up to two mismatches with the on-target sequence. Potential off-target sites were then examined for the presence of a NGG protospacer adjacent motif (PAM) sequence immediately 3′ of the identified protospacer off-target. Only a single off-target site (5′-CGCCGAGGGCGACTACCGGC-3′) was identified using these search criteria. It contained a 2 bp mismatch with the MS45 protospacer target and an AGG PAM (Table 1). To confirm the site was cleaved *in vivo*, it was analysed by deep sequencing for the presence of mutations in maize embryos transformed with DNA vectors expressing Cas9 and gRNA targeting the *MS45* gene. As shown in Table 1, mutational activity was recovered at a frequency of ∼50% of that observed for the on-target site, thus, validating it as an authentic off-target site.

### Data availability

The authors declare that all data supporting the findings of this study are available within the article and its Supplementary Information files or are available from the corresponding author upon request.

## Additional information

**How to cite this article:** Svitashev, S. *et al*. Genome editing in maize directed by CRISPR–Cas9 ribonucleoprotein complexes. *Nat. Commun.* **7**, 13274 doi: 10.1038/ncomms13274 (2016).

**Publisher's note:** Springer Nature remains neutral with regard to jurisdictional claims in published maps and institutional affiliations.

## Supplementary Material

Supplementary Information Supplementary Tables 1-2 and Supplementary Notes 1-3

## Figures and Tables

**Table 1:** Mutation frequencies at the intended and MS45 off-target sites upon delivery of Cas9 and gRNAs as DNA vectors or RNP complexes into maize immature embryo cells.

**Target site**	Target site sequence with PAM*	**Cas9 only (%)**	**DNA delivery (%)**†	**RNP delivery (%)**†
LIG	GCGTACGCGTACGTGTGAGG	0.004	0.56	0.57
ALS2	GCTGCTCGATTCCGTCCCCATGG	0.020‡	0.51	0.45
MS26	GCACGTACGTCACCATCCCGCCGG	0.004	0.43	0.21
MS45	GGCCGAGGTCGACTACCGGCCGG	0.002	0.34	0.69
MS45 off-site	**C**GCCGAGG**G**CGACTACCGGCAGG	0.002	0.18	0.01

Analysis conducted on embryos collected 2 days after bombardment.

There are three nucleotide sequence differences in the MS45 off-target site as compared with the intended site; two within the spacer are shown in bold and underlined, while the off-site PAM is AGG compared with CGG for the intended MS45 target site.

^*^PAM—protospacer adjacent motif is a 3 nt sequence immediately 3′ of the target site.

^†^Low mutation frequency is related to the fact that, when bombarded, only a small percentage of embryo cells receive Cas9–gRNA components, leaving the majority of the cells untransformed with unmodified DNA sequences.

^‡^Higher mutation frequency in the control sample is due to the CCCC stretch at the cut site leading to potential PCR and sequencing mistakes.

**Table 2:** Frequency of mutated alleles at intended and MS45 off-target sites in T0 plants regenerated after delivery of Cas9 and gRNAs as DNA vectors or RNP complexes.

**Target site**	**Cas9 and gRNA delivery method**	**Plants analysed**	**Plants with mutated alleles**	**Plants with biallelic mutations**
MS45	DNA	940	38 (4.0%)	29 (3.1%)
MS45 off-site	DNA	940	19 (2.0%)	4 (0.4%)
MS45	RNP	1,880	70 (3.7%)	9 (0.5%)
MS45 off-site	RNP	1,880	0 (0.0%)	0 (0.0%)
LIG	RNP	756	73 (9.7%)	7 (0.9%)
MS26	RNP	756	18 (2.4%)	2 (0.3%)
