# Supplementary material for: Genome editing in maize directed by CRISPR–Cas9 ribonucleoprotein complexes
Source: Nat Commun. 2016 Nov 16;7:13274. doi: 10.1038/ncomms13274 (PMC5116081; doi:10.1038/ncomms13274)
Supplement: Supplementary Information — Supplementary Tables 1-2 and Supplementary Notes 1-3 [file ncomms13274-s1.pdf]

Supplementary Table 1. List of primers used in deep sequencing analysis study on embryo tissue.

Target Site	Primer Orientation	Primary PCR Primer Sequence with deep sequencing indexes on forward primer where N represents the index sequence
LIG	Forward	CTACACTCTTTCCCTACACGACGCTCTTCCGATCTNNNNCGCAAATGAGTAGCAGCGCAC
	Reverse	CAAGCAGAAGACGGCATACGAGCTCTTCCGATCTCACCTGCTGGGAATTGTACCGTA
MS26	Forward	CTACACTCTTTCCCTACACGACGCTCTTCCGATCTNNNNTCCTGGAGGACGACGTGCTG
	Reverse	CAAGCAGAAGACGGCATACGAGCTCTTCCGATCTCCGGAAGCTCGCCGCGT
MS45	Forward	CTACACTCTTTCCCTACACGACGCTCTTCCGATCTNNNNGGACCCGTTCGGCCTCAGT
	Reverse	CAAGCAGAAGACGGCATACGAGCTCTTCCGATCTGCCGGCTGGCATTGTCTCTG
ALS2	Forward	CTACACTCTTTCCCTACACGACGCTCTTCCGATCTNNNNGTCTGCATCGCCACCTCCGG
	Reverse	CTACACTCTTTCCCTACACGACGCTCTTCCGATCTTTCCGTCTGCATCGCCACCTCCGG
**Target Site**	**Primer Orientation**	**Secondary PCR Primer Sequence**
Universal Primers	Forward	AATGATACGGCGACCACCGAGATCTACACTCTTTCCCTACACG
	Reverse	CAAGCAGAAGACGGCATA

Supplementary Table 2. List of primers used in deep sequencing analysis study on plant tissue.

Target Site	Primer Orientation	Primary PCR Primer Sequence
LIG	Forward	ATCGGGAAGCTGAAGCACCTGCTGGGAATTGTACCGTA
	Reverse	ATCCGACGGTAGTGTCGCAAATGAGTAGCAGCGCAC
MS26	Forward	ATCGGGAAGCTGAAGTCCTGGAGGACGACGTGCTG
	Reverse	ATCCGACGGTAGTGTCCGGAAGCTCGCCGCGT
MS45	Forward	ATCGGGAAGCTGAAGGGACCCGTTCGGCCTCAGT
	Reverse	ATCCGACGGTAGTGTGCCGGCTGGCATTGTCTCTG
ALS2	Forward	ATCCGACGGTAGTGTGTCTGCATCGCCACCTCCGG
	Reverse	ATCGGGAAGCTGAAGCTCCTGGAAGGCGTCGGTGC
MS45 off-site	Forward	ATCGGGAAGCTGAAGGAAGTACCCGCCGGACG
	Reverse	GCCAATTGTGCTATAATTTGTCACCTA
**Target Site**	**Primer Orientation**	**Secondary PCR Primer Sequence with deep sequencing indexes on the reverse primer where N represents the index sequence**
Universal Primers	Forward	AATGATACGGCGACCACCGAGATCTACACATACGAGATCCGTAATCGGGAAGCTGAAG
	Reverse	CAAGCAGAAGACGGCATACGAGATNNNNNNNNACACGCACGATCCGACGGTAGTGT

Supplementary Note 1. Maize Optimized Cas9

ATGGCACCGAAGAAGAAGCGCAAGGTGATGGACAAGAAGTACAGCATCGGCCTCGACATCGGCACCAACTCGGTGGG
CTGGGCCGTCATCACGGACGAATATAAGGTCCCGTCGAAGAAGTTCAAGGTCCTCGGCAATACAGACCGCCACAGCA
TCAAGAAAAACTTGATCGGCGCCCTCCTGTTCGATAGCGGCGAGACCGCGGAGGCGACCAGGCTCAAGAGGACCGCC
AGGAGACGGTACACTAGGCGCAAGAACAGGATCTGCTACCTGCAGGAGATCTTCAGCAACGAGATGGCGAAGGTGGA
CGACTCCTTCTTCCACCGCCTGGAGGAATCATTCCTGGTGGAGGAGGACAAGAAGCATGAGCGGCACCCAATCTTCG
GCAACATCGTCGACGAGGTAAGTTTCTGCTTCTACCTTTGATATATATATAATAATTATCATTAATTAGTAGTAATA
TAATATTTCAAATATTTTTTTCAAAATAAAAGAATGTAGTATATAGCAATTGCTTTTCTGTAGTTTATAAGTGTGTA
TATTTTAATTTATAACTTTTCTAATATATGACCAAAACATGGTGATGTGCAGGTGGCCTACCACGAGAAGTACCCGA
CAATCTACCACCTCCGGAAGAAACTGGTGGACAGCACAGACAAGGCGGACCTCCGGCTCATCTACCTTGCCCTCGCG
CATATGATCAAGTTCCGCGGCCACTTCCTCATCGAGGGCGACCTGAACCCGGACAACTCCGACGTGGACAAGCTGTT
CATCCAGCTCGTGCAGACGTACAATCAACTGTTCGAGGAGAACCCCATAAACGCTAGCGGCGTGGACGCCAAGGCCA
TCCTCTCGGCCAGGCTCTCGAAATCAAGAAGGCTGGAGAACCTTATCGCGCAGTTGCCAGGCGAAAAGAAGAACGGC
CTCTTCGGCAACCTTATTGCGCTCAGCCTCGGCCTGACGCCGAACTTCAAATCAAACTTCGACCTCGCGGAGGACGC
CAAGCTCCAGCTCTCAAAGGACACCTACGACGACGACCTCGACAACCTCCTGGCCCAGATAGGAGACCAGTACGCGG
ACCTCTTCCTCGCCGCCAAGAACCTCTCCGACGCTATCCTGCTCAGCGACATCCTTCGGGTCAACACCGAAATTACC
AAGGCACCGCTGTCCGCCAGCATGATTAAACGCTACGACGAGCACCATCAGGACCTCACGCTGCTCAAGGCACTCGT
CCGCCAGCAGCTCCCCGAGAAGTACAAGGAGATCTTCTTCGACCAATCAAAAAACGGCTACGCGGGATATATCGACG
GCGGTGCCAGCCAGGAAGAGTTCTACAAGTTCATCAAACCAATCCTGGAGAAGATGGACGGCACCGAGGAGTTGCTG
GTCAAGCTCAACAGGGAGGACCTCCTCAGGAAGCAGAGGACCTTCGACAACGGCTCCATCCCGCATCAGATCCACCT
GGGCGAACTGCATGCCATCCTGCGGCGCCAGGAGGACTTCTACCCGTTCCTGAAGGATAACCGGGAGAAGATCGAGA
AGATCTTGACGTTCCGCATCCCATACTACGTGGGCCCGCTGGCTCGCGGCAACTCCCGGTTCGCCTGGATGACCCGG
AAGTCGGAGGAGACCATCACACCCTGGAACTTTGAGGAGGTGGTCGATAAGGGCGCTAGCGCTCAGAGCTTCATCGA
GCGCATGACCAACTTCGATAAAAACCTGCCCAATGAAAAAGTCCTCCCCAAGCACTCGCTGCTCTACGAGTACTTCA
CCGTGTACAACGAGCTCACCAAGGTCAAATACGTCACCGAGGGCATGCGGAAGCCGGCGTTCCTGAGCGGCGAGCAG
AAGAAGGCGATAGTGGACCTCCTCTTCAAGACCAACAGGAAGGTGACCGTGAAGCAATTAAAAGAGGACTACTTCAA
GAAAATAGAGTGCTTCGACTCCGTGGAGATCTCGGGCGTGGAGGATCGGTTCAACGCCTCACTCGGCACGTATCACG
ACCTCCTCAAGATCATTAAAGACAAGGACTTCCTCGACAACGAGGAGAACGAGGACATCCTCGAGGACATCGTCCTC
ACCCTGACCCTGTTCGAGGACCGCGAAATGATCGAGGAGAGGCTGAAGACCTACGCGCACCTGTTCGACGACAAGGT
CATGAAACAGCTCAAGAGGCGCCGCTACACTGGTTGGGGAAGGCTGTCCCGCAAGCTCATTAATGGCATCAGGGACA
AGCAGAGCGGCAAGACCATCCTGGACTTCCTCAAGTCCGACGGGTTCGCCAACCGCAACTTCATGCAGCTCATTCAC
GACGACTCGCTCACGTTCAAGGAAGACATCCAGAAGGCACAGGTGAGCGGGCAGGGTGACTCCCTCCACGAACACAT
CGCCAACCTGGCCGGCTCGCCGGCCATTAAAAAGGGCATCCTGCAGACGGTCAAGGTCGTCGACGAGCTCGTGAAGG
TGATGGGCCGGCACAAGCCCGAAAATATCGTCATAGAGATGGCCAGGGAGAACCAGACCACCCAAAAAGGGCAGAAG
AACTCGCGCGAGCGGATGAAACGGATCGAGGAGGGCATTAAAGAGCTCGGGTCCCAGATCCTGAAGGAGCACCCCGT
GGAAAATACCCAGCTCCAGAATGAAAAGCTCTACCTCTACTACCTGCAGAACGGCCGCGACATGTACGTGGACCAGG
AGCTGGACATTAATCGGCTATCGGACTACGACGTCGACCACATCGTGCCGCAGTCGTTCCTCAAGGACGATAGCATC

GACAACAAGGTGCTCACCCGGTCGGATAAAAATCGGGGCAAGAGCGACAACGTGCCCAGCGAGGAGGTCGTGAAGAA
GATGAAAAACTACTGGCGCCAGCTCCTCAACGCGAAACTGATCACCCAGCGCAAGTTCGACAACCTGACGAAGGCGG
AACGCGGTGGCTTGAGCGAACTCGATAAGGCGGGCTTCATAAAAAGGCAGCTGGTCGAGACGCGCCAGATCACGAAG
CATGTCGCCCAGATCCTGGACAGCCGCATGAATACTAAGTACGATGAAAACGACAAGCTGATCCGGGAGGTGAAGGT
GATCACGCTGAAGTCCAAGCTCGTGTCGGACTTCCGCAAGGACTTCCAGTTCTACAAGGTCCGCGAGATCAACAACT
ACCACCACGCCCACGACGCCTACCTGAATGCGGTGGTCGGGACCGCCCTGATCAAGAAGTACCCGAAGCTGGAGTCG
GAGTTCGTGTACGGCGACTACAAGGTCTACGACGTGCGCAAAATGATCGCCAAGTCCGAGCAGGAGATCGGCAAGGC
CACGGCAAAATACTTCTTCTACTCGAACATCATGAACTTCTTCAAGACCGAGATCACCCTCGCGAACGGCGAGATCC
GCAAGCGCCCGCTCATCGAAACCAACGGCGAGACGGGCGAGATCGTCTGGGATAAGGGCCGGGATTTCGCGACGGTC
CGCAAGGTGCTCTCCATGCCGCAAGTCAATATCGTGAAAAAGACGGAGGTCCAGACGGGCGGGTTCAGCAAGGAGTC
CATCCTCCCGAAGCGCAACTCCGACAAGCTCATCGCGAGGAAGAAGGATTGGGACCCGAAAAAATATGGCGGCTTCG
ACAGCCCGACCGTCGCATACAGCGTCCTCGTCGTGGCGAAGGTGGAGAAGGGCAAGTCAAAGAAGCTCAAGTCCGTG
AAGGAGCTGCTCGGGATCACGATTATGGAGCGGTCCTCCTTCGAGAAGAACCCGATCGACTTCCTAGAGGCCAAGGG
ATATAAGGAGGTCAAGAAGGACCTGATTATTAAACTGCCGAAGTACTCGCTCTTCGAGCTGGAAAACGGCCGCAAGA
GGATGCTCGCCTCCGCAGGCGAGTTGCAGAAGGGCAACGAGCTCGCCCTCCCGAGCAAATACGTCAATTTCCTGTAC
CTCGCTAGCCACTATGAAAAGCTCAAGGGCAGCCCGGAGGACAACGAGCAGAAGCAGCTCTTCGTGGAGCAGCACAA
GCATTACCTGGACGAGATCATCGAGCAGATCAGCGAGTTCTCGAAGCGGGTGATCCTCGCCGACGCGAACCTGGACA
AGGTGCTGTCGGCATATAACAAGCACCGCGACAAACCAATACGCGAGCAGGCCGAAAATATCATCCACCTCTTCACC
CTCACCAACCTCGGCGCTCCGGCAGCCTTCAAGTACTTCGACACCACGATTGACCGGAAGCGGTACACGAGCACGAA
GGAGGTGCTCGATGCGACGCTGATCCACCAGAGCATCACAGGGCTCTATGAAACACGCATCGACCTGAGCCAGCTGG
GCGGAGACAAGAGACCACGGGACCGCCACGATGGCGAGCTGGGAGGCCGCAAGCGGGCAAGGTAG

Supplementary Note 2. Guide RNA expression cassette. Expression cassette containing ZmPolIII promoter, LIG target site spacer (underlined), gRNA (red font) and PolIII terminator.

TGAGAGTACAATGATGAACCTAGATTAATCAATGCCAAAGTCTGAAAAATGCACCCTCAGTCTATGATCCAGAAAAT
CAAGATTGCTTGAGGCCCTGTTCGGTTGTTCCGGATTAGAGCCCCGGATTAATTCCTAGCCGGATTACTTCTCTAAT
TTATATAGATTTTGATGAGCTGGAATGAATCCTGGCTTATTCCGGTACAACCGAACAGGCCCTGAAGGATACCAGTA
ATCGCTGAGCTAAATTGGCATGCTGTCAGAGTGTCAGTATTGCAGCAAGGTAGTGAGATAACCGGCATCATGGTGCC
AGTTTGATGGCACCATTAGGGTTAGAGATGGTGGCCATGGGCGCATGTCCTGGCCAACTTTGTATGATATATGGCAG
GGTGAATAGGAAAGTAAAATTGTATTGTAAAAAGGGATTTCTTCTGTTTGTTAGCGCATGTACAAGGAATGCAAGTT
TTGAGCGAGGGGGCATCAAAGATCTGGCTGTGTTTCCAGCTGTTTTTGTTAGCCCCATCGAATCCTTGACATAATGA
TCCCGCTTAAATAAGCAACCTCGCTTGTATAGTTCCTTGTGCTCTAACACACGATGATGATAAGTCGTAAAATAGTG
GTGTCCAAAGAATTTCCAGGCCCAGTTGTAAAAGCTAAAATGCTATTCGAATTTCTACTAGCAGTAAGTCGTGTTTA
GAAATTATTTTTTTATATACCTTTTTTCCTTCTATGTACAGTAGGACACAGTGTCAGCGCCGCGTTGACGGAGAATA
TTTGCAAAAAAGTAAAAGAGAAAGTCATAGCGGCGTATGTGCCAAAAACTTCGTCACAGAGAGGGCCATAAGAAACA
TGGCCCACGGCCCAATACGAAGCACCGCGACGAAGCCCAAACAGCAGTCCGTAGGTGGAGCAAAGCGCTGGGTAATA
CGCAAACGTTTTGTCCCACCTTGACTAATCACAAGAGTGGAGCGTACCTTATAAACCGAGCCGCAAGCACCGAATTG
CGTACGCGTACGTGTGGTTTTAGAGCTAGAAATAGCAAGTTAAAATAAGGCTAGTCCGTTATCAACTTGAAAAAGTG
GCACCGAGTCGGTGCTTTTTTTTT

Supplementary Note 3. Cas9 protein sequence with 2 NLSs (underlined)

MAPKKKRKVMDKKYSIGLDIGTNSVGWAVITDEYKVPSKKFKVLGNTDRHSIKKNLIGALLFDSGETAEATRLKRTA
RRRYTRRKNRICYLQEIFSNEMAKVDDSFFHRLEESFLVEEDKKHERHPIFGNIVDEVAYHEKYPTIYHLRKKLVDS
TDKADLRLIYLALAHMIKFRGHFLIEGDLNPDNSDVDKLFIQLVQTYNQLFEENPINASGVDAKAILSARLSKSRRL
ENLIAQLPGEKKNGLFGNLIALSLGLTPNFKSNFDLAEDAKLQLSKDTYDDDLDNLLAQIGDQYADLFLAAKNLSDA
ILLSDILRVNTEITKAPLSASMIKRYDEHHQDLTLLKALVRQQLPEKYKEIFFDQSKNGYAGYIDGGASQEEFYKFI
KPILEKMDGTEELLVKLNREDLLRKQRTFDNGSIPHQIHLGELHAILRRQEDFYPFLKDNREKIEKILTFRIPYYVG
PLARGNSRFAWMTRKSEETITPWNFEEVVDKGASAQSFIERMTNFDKNLPNEKVLPKHSLLYEYFTVYNELTKVKYV
TEGMRKPAFLSGEQKKAIVDLLFKTNRKVTVKQLKEDYFKKIECFDSVEISGVEDRFNASLGTYHDLLKIIKDKDFL
DNEENEDILEDIVLTLTLFEDREMIEERLKTYAHLFDDKVMKQLKRRRYTGWGRLSRKLINGIRDKQSGKTILDFLK
SDGFANRNFMQLIHDDSLTFKEDIQKAQVSGQGDSLHEHIANLAGSPAIKKGILQTVKVVDELVKVMGRHKPENIVI
EMARENQTTQKGQKNSRERMKRIEEGIKELGSQILKEHPVENTQLQNEKLYLYYLQNGRDMYVDQELDINRLSDYDV
DHIVPQSFLKDDSIDNKVLTRSDKNRGKSDNVPSEEVVKKMKNYWRQLLNAKLITQRKFDNLTKAERGGLSELDKAG
FIKRQLVETRQITKHVAQILDSRMNTKYDENDKLIREVKVITLKSKLVSDFRKDFQFYKVREINNYHHAHDAYLNAV
VGTALIKKYPKLESEFVYGDYKVYDVRKMIAKSEQEIGKATAKYFFYSNIMNFFKTEITLANGEIRKRPLIETNGET
GEIVWDKGRDFATVRKVLSMPQVNIVKKTEVQTGGFSKESILPKRNSDKLIARKKDWDPKKYGGFDSPTVAYSVLVV
AKVEKGKSKKLKSVKELLGITIMERSSFEKNPIDFLEAKGYKEVKKDLIIKLPKYSLFELENGRKRMLASAGELQKG
NELALPSKYVNFLYLASHYEKLKGSPEDNEQKQLFVEQHKHYLDEIIEQISEFSKRVILADANLDKVLSAYNKHRDK
PIREQAENIIHLFTLTNLGAPAAFKYFDTTIDRKRYTSTKEVLDATLIHQSITGLYETRIDLSQLGGDKKKKLKL
